# Vertical profile of atmospheric conductivity that matches Schumann resonance observations

**DOI:** 10.1186/s40064-016-1742-3

**Published:** 2016-02-01

**Authors:** Alexander P. Nickolaenko, Yuri. P. Galuk, Masashi Hayakawa

**Affiliations:** A.Ya. Usikov Institute for Radio-Physics and Electronics of National Academy of Sciences of the Ukraine, 12 Acad. Proskura Street, Kharkiv, 61085 Ukraine; Saint-Petersburg State University, 35 University Ave., Saint-Petersburg, Peterhof, Russia 198504; Hayakawa Institute of Seismo Electromagnetics Co. Ltd., The University of Electro-Communications (UEC) Incubation Center-508, 1-5-1 Chofugaoka, Chofu, Tokyo, 182-8585 Japan; Advanced Wireless Communications Research Center (AWCC), UEC, 1-5-1 Chofugaoka, Chofu, Tokyo, 182-8585 Japan

**Keywords:** Vertical conductivity profile of atmosphere, ELF radio wave propagation constant, Schumann resonance power spectra, Attenuation factor, Man-made ELF radio waves

## Abstract

We introduce the vertical profile of atmospheric conductivity in the range from 2 to 98 km. The propagation constant of extremely low frequency (ELF) radio waves was computed for this profile by using the full wave solution. A high correspondence is demonstrated of the data thus obtained to the conventional standard heuristic model of ELF propagation constant derived from the Schumann resonance records performed all over the world. We also suggest the conductivity profiles for the ambient day and ambient night conditions. The full wave solution technique was applied for obtaining the corresponding frequency dependence of propagation constant relevant to these profiles. By using these propagation constants, we computed the power spectra of Schumann resonance in the vertical electric field component for the uniform global distribution of thunderstorms and demonstrate their close similarity in all the models. We also demonstrate a strong correspondence between the wave attenuation rate obtained for these conductivity profiles and the measured ones by using the ELF radio transmissions.

## Background

The propagation constant, the source–observer distance, and the current moment of a dipole source are necessary in the standard description of sub-ionospheric radio wave propagation at the extremely low frequency band (ELF: 3–3 kHz). The propagation constant plays an especially important role in computations and modeling. Therefore, significant efforts were directed to its precise estimation (see e.g. Nickolaenko and Hayakawa 2002, 2014 and references therein). The commonly accepted heuristic frequency dependence *ν* (*f*) of the propagation constant has been suggested in Ishaq and Jones (1977) based on the vast experimental data collected at a global array of the Schumann resonance observatories. The observation sites were positioned in both the eastern and western hemispheres. According to Ishaq and Jones (1977), the complex propagation constant *ν* (*f*) is found from the following equations:

1 $$v\left( f \right) = \left[ {0.25 + \left( {kaS} \right)^{2} } \right]^{1/2} - 0.5,$$

where *a* is the Earth’s radius in m, *k* is the free space wavenumber in m^−1^, *f* is the frequency in Hz, and the dimensionless complex sine parameter *S* is given as follows:

2 $$S = c/V{-}i \times 5.49 \times \eta /f,$$

3 $$c/V = 1.64{-}0.1759 \times \ln \left( f \right) + 0.01791 \times \left[ {\ln \left( f \right)} \right]^{2} ,$$

4 $$\eta = 0.063 \times f^{0.64} ,$$

*c* is the light velocity of, *V* is the wave phase velocity in m/s both, and *η* accounts for the wave attenuation in the cavity.

A comparison of experimental Schumann resonance data with those computed from Eqs. (1–4) has confirmed the validity of the model by Ishaq and Jones (1977), although some other models are used in the literature suggesting simpler expressions for the *ν*(*f*) dependence (Nickolaenko and Hayakawa 2002, 2014). We use relations (1–4) in what follows as the standard or the reference model.

In the field computations and in the interpretation of experimental data, the knowledge is redundant of the vertical profile of atmospheric conductivity *σ*(*h*). It is sufficient to use the regular expressions for the electromagnetic fields incorporating the propagation constant, the current moment of the field source, and the ionosphere effective height (see e.g. Nickolaenko and Hayakawa 2002, 2014).

However, information on the vertical profile of atmospheric conductivity *σ*(*h*) becomes obligatory when using the direct modeling methods such as finite difference time domain (FDTD) technique or the 2D telegraph equation (2DTE) (Kirillov 1996; Kirillov et al. 1997; Kirillov and Kopeykin 2002; Morente et al. 2003; Pechony and Price 2004; Yang and Pasko 2005). This kind of computations is impossible without knowing a particular vertical profile of air conductivity and the relevant complex permittivity of atmosphere. The range of heights 50–100 km is crucial for the ELF radio propagation, but it is inaccessible by any modern remote sensing. The existing experimental data on the air conductivity within these altitudes are rare and have been usually obtained by the rocket probes. Therefore, one can find only a limited amount of altitude profiles of the air conductivity in the literature. It is significant that none of these profiles provides a realistic frequency dependence of ELF propagation constant as given by Eqs. (1–4).

The objective of our paper is a realistic *σ*(*h*) profile consistent with the Schumann resonance observations. Such a model profile is desirable when modeling the sub-ionospheric radio propagation in the real Earth–ionosphere cavity.

### The air conductivity as a function of altitude

**Fig. 1:**
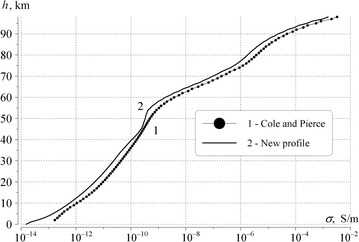
Altitude profiles of air conductivity. *Line 1* is the classic profile (Cole and Pierce 1965); *line 2* is the suggested profile corresponding to Schumann resonance observations in a better way

We start from the classical work (Cole and Pierce 1965) when constructing the altitude dependence *σ*(*h*) corresponding to the observed peak frequencies and the quality factors of the Schumann resonance oscillations. The particular profile *σ*(*h*) in Cole and Pierce (1965) was based on the results of observations and the aeronomy data. This profile is often used in different applications, and it is shown in Fig. 1 by the curve with dots. The major drawback preventing its application in the Schumann resonance studies is inaccurate value of the propagation constant, as seen below. As a result, the computed Schumann resonance spectra noticeably deviate from observations. Our profile (curve 2 in Fig. 1) was obtained from curve 1 by modifications and exhaustive search, and it suggests more realistic data. Simultaneously, it does not seriously deviate from the classical dependence (Cole and Pierce 1965), hence it matches the direct conductivity measurements and the aeronomy data. The particular data on the air conductivity are listed in Table 1.

**Table 1:** Logarithm of air conductivity (S/m) as function of altitude above the ground surface

z km	lg(*σ*)			z km	lg(*σ*)			z km	lg(*σ*)		
	Median	Day	Night		Median	Day	Night		Median	Day	Night
2	−12.77	−12.02	−12.03	34	−10.19	−10.72	−10.73	66	−7.73	−6.62	−9.24
3	−12.68	−11.98	−11.98	35	−10.14	−10.68	−10.69	67	−7.50	−6.39	−9.13
4	−12.60	−11.94	−11.94	36	−10.09	−10.64	−10.65	68	−7.35	−6.16	−9.00
5	−12.51	−11.9	−11.90	37	−10.03	−10.6	−10.6	69	−7.17	−5.94	−8.85
6	−12.43	−11.86	−11.86	38	−10.0	−10.56	−10.56	70	−7.02	−5.71	−8.69
7	−12.31	−11.82	−11.82	39	−9.95	−10.52	−10.52	71	−6.85	−5.48	−8.51
8	−12.22	−11.78	−11.78	40	−9.92	−10.47	−10.48	72	−6.72	−5.25	−8.32
9	−12.08	−11.74	−11.74	41	−9.86	−10.43	−10.44	73	−6.55	−5.02	−8.13
10	−11.97	−11.7	−11.7	42	−9.83	−10.39	−10.40	74	−6.37	−4.79	−7.93
11	−11.84	−11.65	−11.66	43	−9.78	−10.34	−10.36	75	−6.25	−4.56	−7.72
12	−11.74	−11.61	−11.62	44	−9.75	−10.3	−10.32	76	−6.12	−4.34	−7.51
13	−11.62	−11.57	−11.58	45	−9.70	−10.25	−10.28	77	−6.02	−4.11	−7.29
14	−11.53	−11.53	−11.54	46	−9.67	−10.19	−10.24	78	−5.93	−3.88	−7.08
15	−11.42	−11.49	−11.50	47	−9.64	−10.13	−10.2	79	−5.83	−3.65	−6.87
16	−11.34	−11.45	−11.46	48	−9.62	−10.05	−10.16	80	−5.76	−3.42	−6.65
17	−11.25	−11.41	−11.42	49	−9.59	−9.97	−10.12	81	−5.66	−3.19	−6.43
18	−11.17	−11.37	−11.38	50	−9.56	−9.86	−10.08	82	−5.58	−2.96	−6.22
19	−11.09	−11.33	−11.34	51	−9.52	−9.77	−10.04	83	−5.49	−2.73	−6.0
20	−11.02	−11.29	−11.30	52	−9.48	−9.6	−9.99	84	−5.40	−2.51	−5.78
21	−10.94	−11.25	−11.25	53	−9.44	−9.43	−9.96	85	−5.29	−2.28	−5.57
22	−10.88	−11.21	−11.21	54	−9.40	−9.26	−9.91	86	−5.19	−2.05	−5.35
23	−10.80	−11.17	−11.17	55	−9.30	−9.06	−9.87	87	−5.05	−1.82	−5.13
24	−10.74	−11.13	−11.13	56	−9.23	−8.86	−9.83	88	−4.94	−1.59	−4.91
25	−10.67	−11.09	−11.09	57	−9.11	−8.65	−9.79	89	−4.77	−1.36	−4.7
26	−10.61	−11.05	−11.05	58	−9.02	−8.43	−9.74	90	−4.64	−1.14	−4.48
27	−10.55	−11.01	−11.01	59	−8.87	−8.21	−9.7	91	−4.43	−0.91	−4.26
28	−10.49	−10.96	−10.96	60	−8.75	−7.98	−9.65	92	−4.29	−0.68	−4.05
29	−10.42	−10.92	−10.93	61	−8.57	−7.76	−9.60	93	−4.04	−0.45	−3.83
30	−10.37	−10.88	−10.89	62	−8.45	−7.53	−9.55	94	−3.89	−0.22	−3.61
31	−10.32	−10.84	−10.85	63	−8.24	−7.30	−9.48	95	−3.58	0.01	−3.40
32	−10.28	−10.80	−10.81	64	−8.10	−7.08	−9.41	96	−3.40	0.24	−3.18
33	−10.24	−10.76	−10.77	65	−7.87	−6.85	−9.33	97	−3.01	0.46	−2.96
								98	−2.81	0.69	−2.74

The profiles of atmospheric conductivity are shown in Fig. 1 for the altitudes ranging from 0 to 100 km. The thin curve with points 1 shows the classic profile (Cole and Pierce 1965) and the smooth thick curve 2 depicts the more realistic profile *σ*(*h*). As might be seen from the figure, the both curves are rather close to each other, although profile 2 has a more pronounced alteration in the 50–60 km interval (the so-called “knee”). Deviations begin from the 30 km altitude, and the profile 2 becomes “elevated” over the classical plot.

The heuristic “knee model” is popular in the modern Schumann resonance studies proposed in the paper by Mushtak and Williams (2002). It might be applied in computations of the propagation constant instead of formulas (1–4). Similarly to previous works (Kirillov 1996; Kirillov et al. 1997; Kirillov and Kopeykin 2002; Greifinger and Greifinger 1978; Nickolaenko and Rabinowicz 1982, 1987; Sentman 1990a, b; Fullekrug 2000), the knee model postulates a set of parameters allowing computing the two complex characteristic heights (the “electric” and “magnetic” heights) together with the real (i.e., having no imaginary part) scale heights nearby these altitudes. The propagation constant is computed by substituting these parameters into the “standard” equations, while the frequency dependence is postulated for all the model parameters in Mushtak and Williams (2002). After finding the appropriate propagation constant, one can turn to the field computations (Nickolaenko and Hayakawa 2014; Williams et al. (2006)).

Unfortunately, all the works applying the knee model are based on only the verbal description of the relevant *σ*(*h*) profile. None of these depicts the conductivity profile nearby the both characteristic heights. Obtaining such a profile is not a simple task, provided that it is possible at all, especially because all the model parameters depend on the signal frequency. Thus, it is not clear in what a way the real function of height *σ*(*h*), being independent of frequency, might be constructed from the complex functions of frequency. At any rate, the problem remains currently unresolved.

The simplified conductivity profiles are widely used in the direct methods of field computation. These are typically the lg[*σ*(*h*)] plot incorporating the two straight lines that form a twist at the knee altitude due to the change in the scale height (see e.g. Morente et al. 2003; Yang and Pasko 2005; Toledo-Redondo et al. 2013; Molina-Cuberos et al. 2006; Zhou et al. 2013). The vicinity of upper, “magnetic” characteristic height is ignored. The curved height dependence of the air conductivity is in fact the well-known two-scale exponential model. Advantages and drawbacks of such a model are quite well known, and these were comprehensively discussed in the literature (Mushtak and Williams 2002; Sentman 1990a, b; Greifinger et al. 2007). Besides, the two-scale exponential model does not predict any correct values of the peak frequencies and the Q-factors of the Schumann resonance modes when applied in the FDTD technique.

### The propagation constant

The ELF propagation constant *ν* (*f*) is usually constructed on the assumption that the ionosphere plasma is isotropic and horizontally homogeneous. Then, by using the full wave solution (see Hynninen and Galuk 1972; Bliokh et al. 1997, 1980; Galuk and Ivanov 1978; Galuk et al. 2015), one might compute the *ν* (*f*) dependence corresponding to a given profile *σ* (*h*). The full wave solution is the rigorous solution of the radio propagation problem within the vertically stratified ionosphere, and it allows us to obtain the sub-ionospheric propagation constant (*f*). We will mention the major steps in obtaining the solution without reproducing equations here, as these could be found in the above-cited works. The upward and downward waves are taken into account in every plasma layer. Their thickness is much smaller than the wavelength in the medium. The tangential field components are continuous at the layer boundaries. It might be shown then (Hynninen and Galuk 1972; Bliokh et al. 1997; Galuk and Ivanov 1978; Galuk et al. 2015) that the electromagnetic problem is reduced to a nonlinear differential equation of the first order for the surface impedance (the ratio of the tangential components of E and H fields). The surface impedance satisfies boundary conditions at the ground and at the upper boundary in the ionosphere from where the plasma density is supposed to remain constant. The problem is solved numerically by using the iteration procedure, and the desired propagation constant *ν* (*f*) is obtained as a result. The method is regarded as the full wave solution, since it strictly accounts for all the fields propagating in the stratified plasma and in the air.

**Fig. 2:**
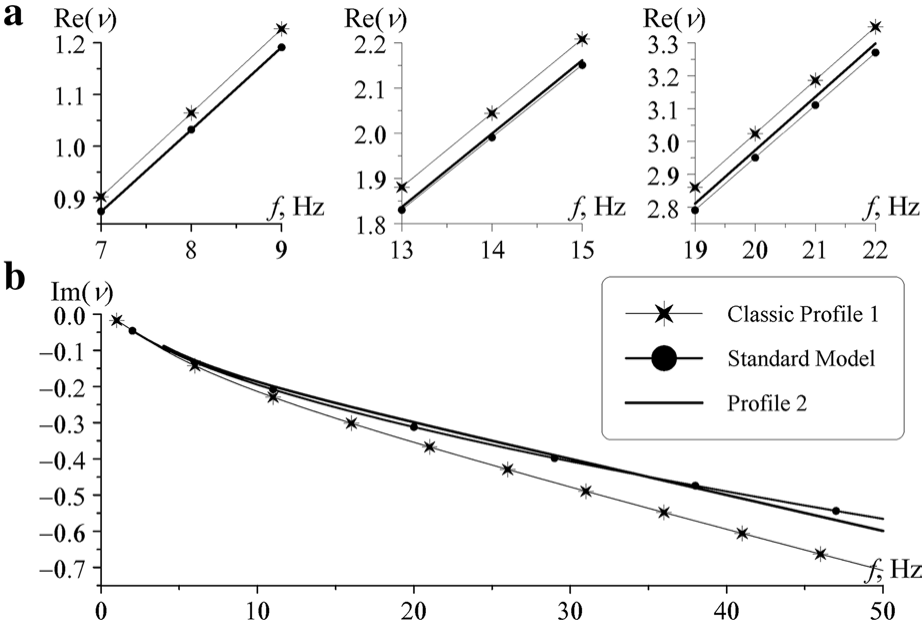
Dispersion curves (Panel a, real part and Panel b, imaginary part). **a** Frequency variations of the real part of the propagation constant: *Lines 1–3* show correspondingly the Re[*ν* (*f*)] functions for the reference model (Ishaq and Jones 1977), for the classical profile (Cole and Pierce 1965), and for the conductivity profile suggested in this paper. **b** The imaginary part of the propagation constant: *line 4* is the model (Ishaq and Jones 1977), *line 5* is the classic profile (Cole and Pierce 1965), and *line 6* is our profile

Frequency variations of the real and imaginary part of the propagation constant are compared in Fig. 2 computed from the formulas (1–4) and from the full wave solution for the profiles 1 and 2 of Fig. 1. Iterations in the full wave solution were performed until the novel value of the surface impedance deviates from the previous one by less than 10^−7^.

As might be seen, all models give very close values in the real part of the propagation constant (i.e., the phase velocity of radio waves), because deviations are only a few percents. So, the resonant frequencies are almost coincident for all three models. But, deviations in the imaginary part or in the attenuation rate of radio waves are more distinct. The standard or the reference model (Ishaq and Jones 1977) and the conductivity profile 2 provide similar dependences (curves 4 and 6), while the attenuation factor following from the classical conductivity profile (Cole and Pierce 1965) (curve 5) considerably deviates from them.

**Fig. 3:**
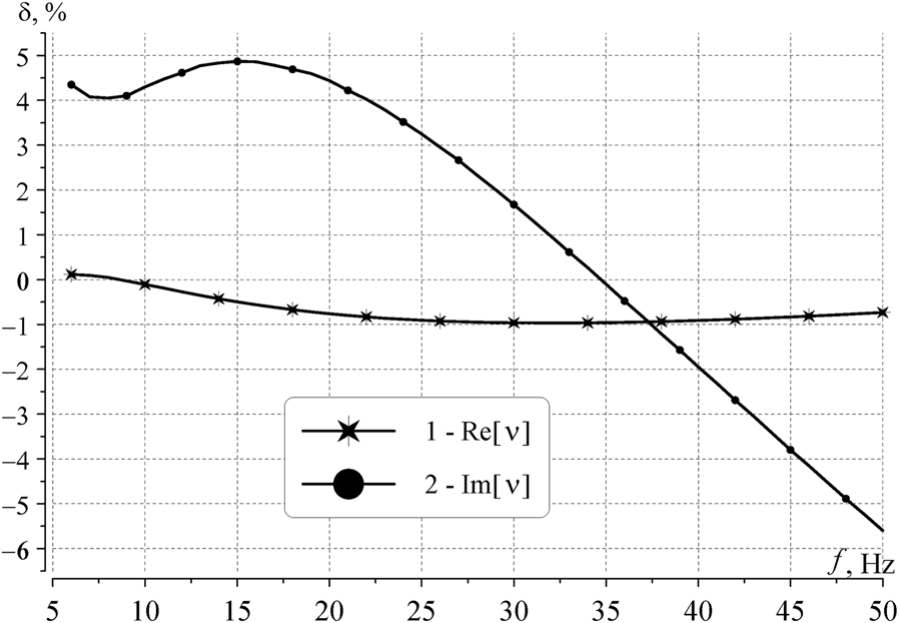
Normalized deviations from the reference model. Normalized deviations from the reference model in the real (*curve 1*) and the imaginary parts (*curve 2*) of the propagation constant computed for profile 2

The normalized deviations are shown in Fig. 3 of the real (curve 1) and the imaginary part of propagation constant (curve 2). Deviations in the real part of the propagation constant were computed from Eq. (5), and excursions of the imaginary part are described by Eq. (6):

5 $$\delta_{R} = 100 \times \{\hbox{Re}[\nu_{2} (f)] - \hbox{Re}[\nu_{0} (f)]\}\hbox{Re}[\nu_{0} (f)],$$

6 $$\delta_{I} = 100 \times \{\hbox{Im}[\nu_{2} (f)] -\{\hbox{Im}[\nu_{0} (f)]\}\hbox{Im} [\nu_{0} (f)].$$

Here *ν*_0_(*f*) is the reference dependence determined from Eqs. (1–4) and *ν*_2_(*f*) is the propagation constant found for profile 2 by using the full wave solution.

Plots in Fig. 3 indicate that profile 2 provides a rather good propagation constant being close to the reference model in the entire Schumann resonance band: deviations in the phase velocity do not exceed 1 %, and those in the attenuation rate are still within an interval of ±5 %. Therefore, profile 2 might be used in modeling of the global electromagnetic resonance of the Earth–ionosphere cavity, especially, in direct methods of field computations, such as FDTD and 2DTE (the parameters are listed in Table 1).

Validity of the conductivity profile #2 might be illustrated also by comparing the computed wave attenuation rate with the data of direct measurements, which was based on the monochromatic radio signals from the ELF transmitters (Bannister 1999; Nickolaenko 2008a, b). Data from the paper by Bannister (1999) were based on the amplitude monitoring of the signal arriving from the US Navy transmitter regarded as the Wisconsin Test Facility (WTF), in which the global network was used to receive the signal. Data were obtained at the frequency of 76 Hz, and the average attenuation rate was 0.82 dB/1000 km for the ambient night and 1.33 dB/1000 km in the ambient day conditions. The average attenuation at this frequency was equal to 1.08 dB/1000 km, and the relative standard deviation due to seasonal variations was ±25 %.

The imaginary part of the propagation constant at this frequency for the profile 2 is equal to Im[*ν* (*f*)]∣_*f*=76_ = 0.86, and this value corresponds to the attenuation *α* (76 Hz) = 1.17 dB/1000 km. This attenuation is practically coincident with that by observations, and this fact is certainly in favor of the model.

The imaginary part of the propagation constant was also published in the papers (Nickolaenko 2008a, b), and it was measured at the 82 Hz frequency. It is equal to Im[*ν* (*f*)]∣_*f*=82_ = 0.92, which corresponds to the attenuation factor *α* (76 Hz) = 1.25 dB/1000 km. The attenuation rate in Nickolaenko (2008a) was inferred from the distance dependence of the signal amplitude in the vertical electric field components while the radio wave was emitted from the Kola Transmitter of the Soviet Navy. The model imaginary part of the propagation constant Im[*ν* (*f*)]∣_*f*=82_ = 0.92 is equal to the value measured experimentally.

A comparison with observations of the man-made ELF radio transmissions justifies the employment of the conductivity profile #2 in ELF applications.

### Comparison of the power spectra

**Fig. 4:**
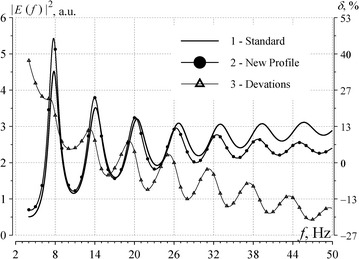
Frequency spectra of computed vertical electric fields. Schumann resonance spectra in the vertical electric field computed for the uniform global distribution of thunderstorms. The smooth *curve 1* is the power spectrum obtained in the reference model. *Curve 2* (*with dots*) shows the similar spectrum relevant to profile 2 of Fig. 1. *Line 3* (relevant to the right ordinate) depicts deviations (in %) from the reference *curve*

The major goal of constructing propagation constant is its further application in the field computations. To demonstrate similarity of the results obtained with the profile 2 and the reference model (Ishaq and Jones 1977), we plot the power spectra of the vertical electric field in Fig. 4. The globally uniform spatial distribution of the sources was used in Williams et al. (2006) for eliminating the possible influence of the source–observer distance on the spectrum outline. In the case of uniform source distribution, the power spectrum is described by the following equation (Nickolaenko and Hayakawa 2002, 2014):

7 $$\left| {E\left( f \right)} \right|^{2} \approx \left| {Ids\left( \omega \right)\frac{{\nu \left( {\nu + 1} \right)}}{\omega }} \right|^{2} \sum\limits_{n = 0}^{\infty } {\frac{2n + 1}{{\left| {n\left( {n + 1} \right) - \nu \left( {\nu + 1} \right)} \right|^{2} }}} .$$

Here, *ω* = 2*π* *f* is the circular frequency, *n* = 1, 2, 3, … is the Schumann resonance mode number, and *Ids*(*ω*) is the current moment of the field source being constant within the ELF band.

Two resonance spectra are shown in Fig. 4. The smooth line 1 corresponds to the spectrum computed with the reference propagation constant (Ishaq and Jones 1977), and the line with dots 2 is the spectrum relevant to our conductivity profile of the atmosphere. Relative deviations in percents from the reference spectrum are shown by curve 3 relevant to the right ordinate. By comparing Figs. 3 and 4, we observe that deviations in the spectra are more apparent than in the dispersion curves *ν* (*f*). Even a difference in the phase velocity of about 1–2 % is clearly visible in the spectra: the peak frequencies of the higher modes noticeably diverge. Curve 3 in Fig. 4 illustrates that relative deviations of the power spectrum occupy the interval from −5 to +15 %, and this is 3–4 times smaller than deviations pertinent to the classical profile (Ishaq and Jones 1977).

### Accounting for ambient day and night conditions

**Fig. 5:**
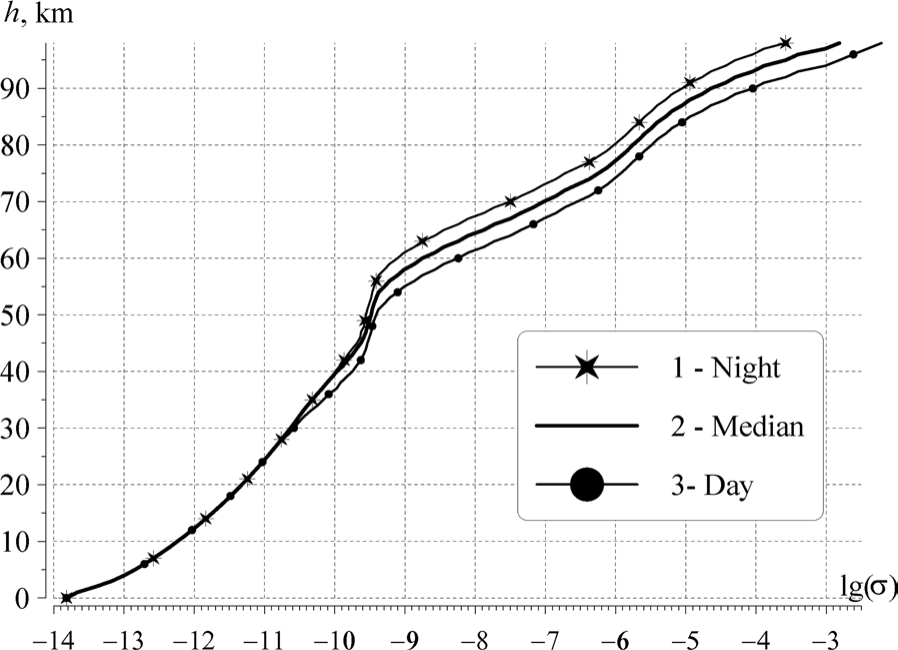
Vertical profiles of atmospheric conductivity. *Curve 1* corresponds to ambient night conditions; profile 3 is relevant to the ambient day conditions; *line 2* is the median profile

The conductivity profile #2 is consistent with the Schumann resonance observations and with measurements of attenuation rate of man-made ELF radio waves. This allows us to proceed further and to introduce the *σ*(*h*) profiles for the ambient day and ambient night conditions. The corresponding graphs are shown in Fig. 5.

The horizontal axis of Fig. 5 depicts the logarithm of air conductivity, and the vertical axis is the altitude above the ground. The smooth curve 2 reproduces the *σ*(*h*) profile that was shown by line 2 in Fig. 1. Line 1 in Fig. 5 corresponds to the conductivity at ambient night, i.e. when the ionosphere is known to be higher than by day. The curve 3 corresponds to ambient day condition.

By using the full wave solution, we computed the frequency dependence of the complex propagation constant for the day and the night profiles, and compared these data with the reference model of *ν*(*f*). When propagation constant is known, one can compute the power spectra of resonance oscillations in the ambient day and ambient night conditions. We are not going to investigate the effect of the ionosphere day–night asymmetry on the global electromagnetic resonance. Therefore, the term “ambient day condition” means that the horizontally uniform ionosphere is described by the day profile all over the globe. Similarly, the words “ambient night condition” mean in what follows that the night profile of the ionosphere is valid over all points of the Earth’s surface.

**Fig. 6:**
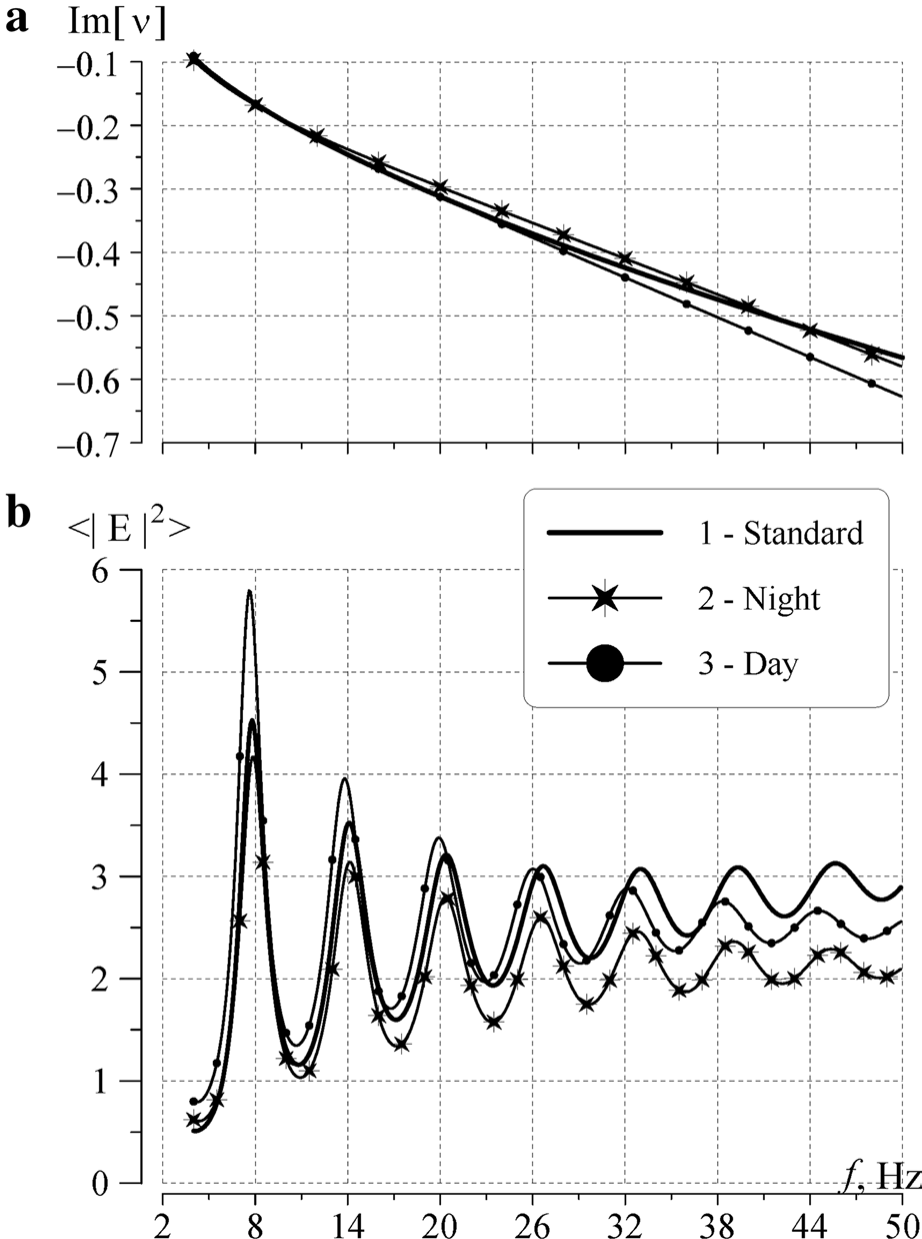
Attenuation rates of ELF waves and power spectra of vertical electric field. **a** Frequency variations of the imaginary part of propagation constant. *Line 1* is the reference dependence (1)–(4); *lines 2* and *3* characterize losses in the “whole day” and the “whole night” cavities. **b** Power spectra of the vertical electric field. *Line 1* is the reference spectrum obtained with the propagation constant (1)–(4); *lines 2* and *3* show spectra for the day and night conductivity profiles

Again, to eliminate the influence of the source–observer distance we use the uniform global distribution of thunderstorms being sources of Schumann resonance. Obviously, the “day” and the “night” spectra thus obtained will correspond to two ultimate provisional situations of the “complete day” or the “complete night” ionosphere in the resonator. The spectrum pertinent to a realistic cavity with the day–night inhomogeneity should occupy an intermediate position between these two extreme curves (see Fig. 6).

Figure 6 shows the computational data for the day and night conductivity profiles. Graphs in the upper panel of Fig. 6 demonstrate that the reference attenuation rate (curve 1) lies between the values obtained for the night (curve 2) and the day (curve 3) conductivity profiles within all Schumann resonance band. The bottom panel of this figure depicts the power spectra of the vertical electric field. As it was expected, the resonance peaks of the “night” power spectrum (curve with stars) are higher than those of the “day” spectrum. The resonance frequencies and the quality factors corresponding to the night conductivity profile are also higher than those corresponding to the daytime ionosphere. The spectrum relevant to the reference propagation constant occupies an intermediate position between the “day” and “night” spectra. Thus, the outline of power spectra confirms the validity of the day and night conductivity profiles that we have shown in Fig. 5 and presented in Table 1.

## Discussion and conclusions

The height profile 2 of atmospheric conductivity is close to the classical profile 1, and it simultaneously agrees with the Schumann resonance parameters. The realistic propagation constant *ν*(*f*) is obtained when one applies the rigorous full wave solution of the electrodynamics problem to the conductivity profile #2. It is rather close to the reference dependence *ν*(*f*) widely used in the literature. Simultaneously, the model values of profile 2 agree with the attenuation rate obtained from the man-made ELF radio transmissions at frequencies above the Schumann resonance (Bannister 1999; Nickolaenko 2008a). We list the corresponding data in Table 2.

**Table 2:** Radio wave attenuation at discrete frequencies obtained from conductivity profile and measured experimentally

Kind of data	〈Im(*ν*)〉	〈*α*〉 dB/1000 km	Im(*ν*) Day	*α* Day dB/1000 km	Im(*ν*) Night	*α* Night dB/1000 km
*f* = 76 Hz model	0.86	1.17	0.96	1.31	0.75	1.02
*f* = 76 Hz experiment (Williams et al. 2006)	–	1.08	–	1.33	–	0.82
*f* = 82 Hz model	0.92	1.25	1.01	1.38	0.79	1.08
*f* = 82 Hz experiment (Yang Pasko 2005; Zhou et al. 2013)	–	1.25	–	–	–	–

Table 2 compares values of attenuation rate of ELF radio waves computed for the conductivity profile presented in Table 1 with those published in the literature and presenting the results of measurements of radio signals from the ELF radio transmitters. Data at 76 Hz were taken from the survey (Bannister 1999), which summarized the long-term observations of the signals transmitted by the US Navy Wisconsin Test Facility.

Data for the frequency of 82 Hz were obtained from the observed distance dependence of the vertical electric field arriving from the Kola Peninsula Soviet Navy ELF transmitter [29, 30]. It is necessary to note that Im (*ν*) is the dimensionless quantity measured in Napier per radian. The experimentally deduced attenuation rate *α* is measured in dB/1000 km. The quantities are connected by the following relation:

8 $$\alpha = \pi \times \hbox{lg}\left( e \right) \cdot \hbox{Im}(\nu ) \approx 1.346 \cdot \hbox{Im}(\nu )$$

The model values Im(*ν*) from Table 2 were translated in accordance with this formula to the equivalent attenuation *α*. As might be seen, the average model attenuation rate at the frequency of 76 Hz is 1.17 dB/1000 km, and the experimentally measured value is 1.08 dB/1000 km. The deviation is about 7 %. Deviation of the model attenuation from that measured in the ambient day and night conditions are equal to 2 % and 24 % correspondingly. The attenuation values at 82 Hz are equal to 1.25 dB/1000 km, and the mutual deviation was less than 1 %. These data lead to the conclusion that the vertical profile 2 of the air conductivity suggested here is justified not only in the frequency band of global electromagnetic resonance, but also at frequencies above it.

We analyzed and compared model results with the literature data available and demonstrated that the suggested vertical profile of the atmospheric conductivity is a rather realistic model. Firstly, it is consistent with the classical concept of the air ionization. Secondly, application of this profile in the full wave solution provides the frequency dependence of the ELF propagation constant close to the reference one in the whole Schumann resonance band. Third, the computed the propagation constant is in good agreement with measurements of the man-made ELF radio signals.

When thinking about areas of future works, we anticipate that our profile will be useful in direct modeling of Schumann resonance: the FDTD algorithms and in the 2DTU approach. In particular, all published FDTD solutions had Schumann resonance frequencies exceeding the observed values. Deviations have arisen from unrealistic conductivity profiles applied in these models. We are sure that profiles presented here will improve the data of direct modeling, and we plan applying the profiles in future investigations of Schumann resonance.

## References

[CR1] Bannister PR (1999). Further examples of seasonal variations of ELF radio propagation parameters. Radio Sci.

[CR2] Bliokh PV, Nickolaenko AP, Filippov YuF (1980). Schumann resonances in the Earth-ionosphere cavity.

[CR3] Bliokh PV, Galuk YuP, Hynninen EM, Nickolaenko AP, Rabinowicz LM (1997). On the resonance phenomena in the Earth-ionosphere cavity. Izv. VUZOV, Radiofizika.

[CR4] Cole RK, Pierce ET (1965). Electrification in the Earth’s atmosphere from altitudes between 0 and 100 kilometers. J Geophys Res.

[CR5] Fullekrug M (2000). Dispersion relation for spherical electromagnetic resonances in the atmosphere. Phys Lett A.

[CR6] Galuk YP, Ivanov VI (1978) Deducing the propagation characteristics of VLF fields in the cavity Earth—non-uniform along the height anisotropic ionosphere. In: Problems of diffraction and radio wave propagation, vol 16. Leningrad State University Press, Leningrad, pp 148–153 **(in Russian)**

[CR7] Galuk YP, Nickolaenko AP, Hayakawa M (2015). Knee model: comparison between heuristic and rigorous solutions for the Schumann resonance problem. J Atmos Sol-Terr Phys.

[CR8] Greifinger C, Greifinger P (1978). Approximate method for determining ELF eigenvalues in the Earth-ionopshere waveguide. Radio Sci.

[CR9] Greifinger PS, Mushtak VC, Williams ER (2007). On modeling the lower characteristic ELF altitude from aeronomical data. Radio Sci..

[CR10] Hynninen EM, Galuk YP (1972) Field of vertical dipole over the spherical Earth with non-uniform along height ionosphere. In: Problems of diffraction and radio wave propagation, vol 11. Leningrad State University Press, Leningrad, pp 109–120 **(in Russian)**

[CR11] Ishaq M, Jones DL (1977). Method of obtaining radiowave propagation parameters for the Earth–ionosphere duct at ELF. Electron Lett.

[CR12] Kirillov VV (1996). 2D theory of ELF electromagnetic wave propagation in the Earth–ionosphere cavity. Izv. VUZOV, Radiofizika.

[CR13] Kirillov VV, Kopeykin VN (2002). Solution of 2D telegraph equations with anisotropic parameters. Izv. VUZOV, Radiofizika.

[CR14] Kirillov VV, Kopeykin VN, Mushtak VC (1997). Electromagnetic waves of ELF band in the Earth–ionosphere cavity. Geomagn Aeron.

[CR15] Molina-Cuberos GJ, Morente JA, Besser BP, Portí J, Lichtenegger H, Schwingenschuh K, Salinas A, Margineda J (2006). Schumann resonances as a tool to study the lower ionospheric structure of Mars. Radio Sci..

[CR16] Morente JA, Molina-Cuberos GJ, Portí JA, Besser BP, Salinas A, Schwingenschuch K, Lichtenegger H (2003). A numerical simulation of Earth’s electromagnetic cavity with the Transmission Line Matrix method: Schumann resonances. J Geophys Res.

[CR17] Mushtak VC, Williams E (2002). Propagation parameters for uniform models of the Earth–ionosphere waveguide. J Atmos Solar Terr Phys.

[CR18] Nickolaenko AP (2008). Deducing the ELF attenuation rate from the distance dependence of radio wave emitted by man-made source. Radio Phys Electron.

[CR19] Nickolaenko AP (2008). ELF attenuation factor derived from distance dependence of radio wave amplitude propagating from an artificial source. Telecommun Radio Eng.

[CR20] Nickolaenko AP, Hayakawa M (2002). Resonances in the Earth–ionosphere cavity.

[CR21] Nickolaenko A, Hayakawa M (2014). Schumann resonance for tyros (Essentials of global electromagnetic resonance in the earth–ionosphere cavity).

[CR22] Nickolaenko AP, Rabinowicz LM (1982). On a possibility of global electromagnetic resonances at the planets of Solar system. Kosm Issled.

[CR23] Nickolaenko AP, Rabinowicz LM (1987). On applicability of ELF global resonances for studying thunderstorm activity at Venus. Kosm Issled.

[CR24] Pechony O, Price C (2004). Schumann resonance parameters computed with a partially uniform knee model on Earth, Venus, Mars, and Titan. Radio Sci.

[CR25] Sentman DD (1990). Approximate Schumann resonance parameters for two-scale-height ionosphere. J Atmos Terr Phys.

[CR26] Sentman DD (1990). Electrical conductivity of Jupiter Shallow interior and the formation of a resonant planetary–ionospheric cavity. Icarus.

[CR27] Toledo-Redondo S, Salinas A, Morente-Molinera JA, Mendez A, Fornieles J, Portí J, Morente JA (2013). Parallel 3D-TLM algorithm for simulation of the Earth–ionosphere cavity. J Comput Phys.

[CR28] Williams ER, Mushtak VC, Nickolaenko AP (2006). Distinguishing ionospheric models using Schumann resonance spectra. J Geophys Res.

[CR29] Yang H, Pasko VP (2005). Three-dimensional finite-difference time domain modeling of the Earth–ionosphere cavity resonances. Geophys Res Lett.

[CR30] Zhou H, Yu H, Cao B, Qiao X (2013). Diurnal and seasonal variations in the Schumann resonance parameters observed at Chinese observatories. J Atmos Solar Terr Phys.

